# Organization of Medical Society of Mercer Co., Ill.

**Published:** 1858-09

**Authors:** J. A. Maury, W. R. Fox

**Affiliations:** President; Secretary


### ORGANIZATION OF MEDICAL SOCIETY OF MERCER CO., ILL.

Pursuant to previous notice, the physicians of Mercer County met at Aledo, August 31st, 1858, to organize a County Medical Society.

On motion, Dr. J. P. Boyd, of Millersburg, was called to the chair; and Dr. O. P. S. Plummer, of Aledo, was chosen Secretary.

On motion, a committee of three, consisting of Drs. Maury, Wood and Allen, were appointed to present a Constitution and By-Laws for said society.

The committee submitted the following Constitution and By-Laws, which, on motion, were approved and adopted, article by article:

CONSTITUTION.

Art. I. This society shall be known and designated the Mercer County Medical Society.

Art. II. The physicians present at the adoption of this Constitution shall be members of the Society.

Art. III. Any regular graduate of an orthodox school, or any physician passing a satisfactory examination before the Board of Censors, may become a member by a vote of the society.

Art. IV. The officers of the society shall consist of a President, Vice-President, Secretary, who shall act as Treasurer, and three Censors; to be elected annually by a majority vote of all the members present.

Art. V. The society shall be governed by the code of ethics adopted by the Illinois State Medical Society.

Art. VI. This Constitution may be altered or amended by a vote of two-thirds of the members present at any regular meeting, by giving notice one meeting previous.

BY-LAWS.

*1st.* The regular meetings of this society shall be the last Wednesday of August, November, February and May, the last of which shall be considered the regular annual meeting.

*2d.* The duties of the officers shall be those that usually pertain to such societies, and all officers shall be elected by ballot at the annual meeting.

*3d.* Each member shall report, in writing, a case, or read an essay at every regular meeting, and a stated essay shall be read at each meeting, the essayist to be appointed at the previous meeting, excepting the annual address, which shall be by the President retiring.

*4th.* The report of cases and essays made before the society at its regular meetings shall become the property of the society.

*5th.* Application being made to the President by three members, he shall, through the Secretary, notify the society of a called meeting.

On motion, the society proceeded to the election of officers, when the following were duly elected:

J. H. MAURY, - - - - - *President.*
J. P. BOYD, - - - - - *Vice-President.*
W. R. FOX, - - - - *Sec. and Treas.*
DRS. WOOD, PLUMMER and BENEDICT, *Censors.*

On motion, Drs. Wood, Plummer and Boyd were appointed to present a Fee Bill at the next meeting.

On motion, Dr. O. P. Allen was appointed to write an essay on Homœopathy, to be read at the next meeting.

The Secretary was instructed to forward the proceedings of this meeting to the *Chicago Medical Journal* and our county papers for publication.

On motion, the society adjourned to meet in Aledo on the last Wednesday of November next.

J. A. MAURY, *President.*

W. R. FOX, *Secretary.*

